# ABCB1 Polymorphisms Influence on Temozolomide Resistance and Overall Survival in Glioblastoma Patients: A Systematic Review of Clinical Evidence

**DOI:** 10.1111/jcmm.71130

**Published:** 2026-04-09

**Authors:** Fabiola De Luca, Deborah Mannino, Anna Paola Capra, Giuliana Ciappina, Marco Donato, Giuseppe Caruso, Emanuela Esposito, Alessio Ardizzone

**Affiliations:** ^1^ Department of Chemical, Biological, Pharmaceutical and Environmental Sciences University of Messina Messina Italy; ^2^ Research Operative Unit of Neuropharmacology and Translational Neurosciences Oasi Research Institute, IRCCS Troina Italy; ^3^ Department of Medical Sciences, Section of Experimental Medicine University of Ferrara Ferrara Italy; ^4^ Division of Medical Oncology, AOU “G.Martino” Hospital University of Messina Messina Italy; ^5^ UniCamillus‐Saint Camillus International University of Health Sciences Rome Italy; ^6^ IRCSS San Camillo Hospital Venice Italy

**Keywords:** ABCB1 polymorphisms, chemoresistance, glioblastoma, overall survival, temozolomide

## Abstract

Glioblastoma (GB), defined as IDH‐wildtype CNS WHO grade 4 tumour according to the 2021 WHO classification of CNS tumours, remains a uniformly lethal malignancy in which the efficacy of temozolomide (TMZ) continues to be constrained by both intrinsic tumur biology and the pharmacological barrier imposed by the blood–brain barrier (BBB). Given the central role of the ABCB1 (MDR1/P‐glycoprotein) efflux transporter in regulating CNS drug disposition, germline variation in ABCB1 has been proposed as a potential determinant of interindividual variability in TMZ response. This systematic review synthesised clinical evidence from four independent studies, encompassing more than 400 GB patients, evaluating the association between ABCB1 polymorphisms and TMZ efficacy and patients' survival. Across the available literature, the influence of ABCB1 genetic variation emerged as limited and inconsistent. An early study reported a marked survival advantage for carriers of the ABCB1 C1236T C/C genotype treated with TMZ, suggesting reduced efflux and enhanced drug exposure. However, subsequent investigations, including epigenetic analyses, high‐quality multivariate survival modelling and a pharmacokinetic study demonstrating genotype‐dependent differences in plasma TMZ concentrations, did not replicate a corresponding survival effect. Across the remaining cohorts, common variants such as 1236C>T, 2677G>T/A, 3435C>T and 1199G>A showed no robust association with clinical outcome, indicating that transporter‐mediated modulation is likely overshadowed by dominant prognostic drivers, including MGMT methylation, IDH status and tumour heterogeneity. Collectively, current evidence does not support ABCB1 polymorphisms as reliable predictive biomarkers of TMZ response in GB. Nonetheless, the pharmacokinetic signals observed, together with emerging technologies capable of selectively modulating efflux activity at the tumour–BBB interface, point to a continued role for ABCB1 in future therapeutic strategies. Integration of transporter genomics with spatial pharmacokinetics and molecular stratification will be essential to refine drug delivery and improve outcomes in GB.

## 1 Introduction

Glioblastoma (GB), defined as IDH‐wildtype CNS WHO grade 4 tumour according to the 2021 WHO classification of central nervous system (CNS) tumours, is the most aggressive primary malignant brain tumour in adults [1]. Hallmark features include rapid proliferation, diffuse infiltration, necrosis and pronounced neo‐angiogenesis [2]. Beyond these histopathological attributes, GB exhibits a highly complex genomic architecture that shapes biological behaviour, therapeutic response and survival [3].

Recurrent alterations include amplification of EGFR in ~40% of cases, frequently accompanied by expression of the constitutively active EGFRvIII variant, which amplifies PI3K/AKT and RAS/MAPK signalling, promoting proliferation, survival and therapeutic escape [4, 5]. Although less frequent, IDH mutations identify a distinct biological entity, astrocytoma, IDH‐mutant, which can also reach CNS WHO grade 4 and is associated with a more favourable prognosis, reflecting metabolic rewiring and epigenetic reprogramming driven by 2‐hydroxyglutarate [6].

Pharmacological treatment of GB relies primarily on temozolomide (TMZ), an oral alkylating agent of the imidazotetrazine class [7]. After administration, TMZ undergoes rapid non‐enzymatic conversion to its active metabolite, 5‐(3‐methyltriazen‐1‐yl)imidazole‐4‐carboxamide (MTIC), which induces cytotoxicity by forming O6‐methylguanine adducts in DNA [8].

The therapeutic effect of TMZ depends on persistence of these lesions through replication, resulting in futile mismatch repair cycles and cytotoxicity [7]. A central biomarker of TMZ benefit is methylation of the MGMT promoter, which silences the direct repair enzyme O6‐methylguanine‐DNA methyltransferase and thereby increases the effective potency of alkylation [9].

To date, no alternative systemic therapy has demonstrated superiority over TMZ in the first‐line setting [10]. Immunotherapeutic strategies, including checkpoint inhibitors and vaccines, remain still under investigation but have yet to show consistent survival improvement [11]. Thus, despite two decades of drug development, TMZ remains the only agent that consistently improves survival when combined with radiotherapy [12].

Although TMZ is comparatively well tolerated relative to legacy nitrosoureas, its therapeutic window in GB remains narrow. Haematological toxicity, neutropenia, thrombocytopenia and cumulative lymphopenia, is dose‐limiting and can force intensity reductions that flatten exposure–response relationships [13]. In addition, gastrointestinal adverse effects are common and often lead to reduced treatment adherence in clinical practice, particularly among elderly or frail patients [14].

In GB therapy, the blood‐brain barrier (BBB) remains a major pharmacological obstacle: structurally heterogeneous, functionally selective and dynamically regulated [15]. TMZ penetration across BBB is further influenced by interindividual variability in plasma protein binding, local pH gradients and efflux transporter activity [16]. As a result, therapeutic drug concentrations in tumour tissue may not always be achieved, even when systemic exposure appears adequate. Thus, the practical challenge with TMZ is twofold: toxicity that restricts the ability to escalate dose and biological/drug‐distribution barriers that prevent achieving effective DNA‐alkylating burden.

In the milieu of molecular determinants that regulate intratumoral drug exposure, ATP‐binding cassette (ABC) transporters play a central role. These efflux systems are strategically expressed at key pharmacological barrier interfaces, including the luminal endothelium of the BBB, choroid plexus, intestinal epithelium, hepatocytes and renal tubular cells, where they actively extrude a wide range of xenobiotics and limit tissue drug accumulation [17]. At the BBB, their polarised localisation at the luminal membrane is critical for maintaining CNS homeostasis by restricting the entry of potentially harmful compounds and facilitating systemic clearance [18].

Among these transporters, ABCG2 (ATP‐binding cassette sub‐family G member 2, also known as breast cancer resistance protein, BCRP) represents a key component of the BBB efflux machinery [19]. Co‐expressed with other transporters and characterised by partially overlapping substrate specificity, ABCG2 contributes to a coordinated and, in some cases, compensatory barrier function that can significantly influence CNS drug disposition [20, 21].

Although TMZ crosses the BBB primarily via passive diffusion owing to its small size and favourable physicochemical properties, its distribution within the CNS may still be modulated by active efflux mechanisms [22].

In this context, ABCB1 (P‐glycoprotein) is of particular relevance. As a high‐capacity, ATP‐dependent efflux transporter encoded by the ABCB1 (MDR1) gene, it exhibits broad substrate promiscuity and plays a dominant role in limiting intracerebral drug accumulation [23, 24]. The expression of ABCB1 at the luminal surface of brain capillary endothelial cells enables vectorial transport of substrates from the brain parenchyma back into the systemic circulation, thereby maintaining steep concentration gradients across the BBB [25]. Beyond its physiological protective role, ABCB1 is a major determinant of pharmacokinetic variability, influencing drug absorption, distribution and elimination across multiple organs [26].

In the oncological setting, this function becomes a critical barrier to effective chemotherapy, as many structurally unrelated anticancer agents are recognised substrates of ABCB1 and its overexpression has been associated with multidrug resistance phenotypes in some cases [27]. Consequently, interindividual variability in ABCB1 expression or function, driven by germline genetic variation, may influence drug penetration across the BBB, intratumoral exposure and ultimately therapeutic response to TMZ [28, 29].

We previously conducted a systematic review on ABCB1 polymorphisms in Familial Mediterranean Fever (FMF), demonstrating that variability in this transporter can influence clinical response to colchicine [30]. These findings reinforced the principle that ABCB1 is not simply a pharmacokinetic modifier, but a clinically actionable character of interindividual variability. Extending this framework to oncology, the present systematic review focuses on GB treated with TMZ, where cytotoxic efficacy depends critically on achieving sufficient intratumoral alkylating burden. Given that ABCB1 is highly expressed at the blood–brain barrier and exhibits broad affinity for small‐molecule chemotherapeutics, polymorphic variation at ABCB1 loci could plausibly alter TMZ penetration, residence time and ultimately clinical outcome.

Therefore, the objective of this review is to systematically evaluate the available clinical evidence regarding the association between ABCB1 variants and TMZ response in patients with GB.

## 2 Methods

### 2.1 Search Strategy

A systematic literature search was carried out across the PubMed (MEDLINE), Embase (via Ovid) and Web of Science databases. The search strategy was designed and executed in accordance with the Preferred Reporting Items for Systematic Reviews and Meta‐Analyses Protocols (PRISMA‐P) recommendations to ensure methodological transparency and reproducibility [31]. Two independent reviewers (D.M. and F.D.L.) performed the screening and selection process, restricting inclusion to studies published in English. Articles were assessed according to predefined eligibility criteria summarised in Table 1.

**TABLE 1:** Summary of inclusion and exclusion criteria applied during the systematic literature search and study selection process.

Inclusion criteria	Exclusion criteria
Clinical trials, randomised controlled trials, observational studies, case–control studies, cross‐sectional studies, cohort studies	Case reports, editorials, letters, reviews, guidelines, abstracts and paper conferences, systematic reviews and meta‐analyses and ongoing studies Articles not written in English

Two domain specialists (A.P.C. and A.A.) supervised both the development of the search strategy and the overall conduct of the review process. The electronic search covered all records available from database inception through October 2025, without applying geographical restrictions. Eligible studies were those investigating the association between the ABCB1 transporter and TMZ therapy in the clinical context of GB.

The specific search strings, outlined in Table 2, combined controlled vocabulary and free‐text terms related to ABCB1, TMZ and GB.

**TABLE 2:** Search query structure and keyword combinations used across databases to identify studies evaluating the relationship between ABCB1, TMZ and GB.

GB	TMZ	ABCB1
‘glioblastoma’ OR ‘glioblastomas’ OR ‘glioblastoma multiforme’ OR ‘GBM’ OR ‘GB’ OR ‘brain tumour’ OR ‘brain tumours’ OR ‘malignant glioma’ OR ‘malignant gliomas’ OR ‘astrocytoma grade IV’ OR ‘astrocytomas grade IV’	‘temozolomide’ OR ‘TMZ’ OR ‘Temodar’ OR ‘Temodal’ OR ‘alkylating agent’ OR ‘alkylating agents’ OR ‘DNA methylating agent’ OR ‘DNA methylating agents’ OR ‘antineoplastic agent’ OR ‘antineoplastic agents’ OR ‘chemotherapy’ OR ‘chemotherapies’ OR ‘chemotherapeutic drug’ OR ‘chemotherapeutic drugs’ OR ‘anticancer drug’ OR ‘anticancer drugs’	‘ABCB1’ OR ‘MDR1’ OR ‘P‐glycoprotein’ OR ‘P‐gp’ OR ‘PGP’ OR ‘ATP‐binding cassette sub‐family B member 1’ OR ‘P‐glycoprotein transporter’ OR ‘Multidrug resistance gene 1’ OR ‘ABC transporter B1’

### 2.2 Study Selection

Relevant studies were identified through systematic searches of PubMed (MEDLINE), Embase (via Ovid) and Web of Science. Duplicate records were removed prior to screening. Two independent reviewers (D.M. and F.D.L.) evaluated titles and abstracts to exclude non‐eligible publications, followed by full‐text assessment of potentially relevant articles according to the predefined inclusion criteria. Any discrepancies between the reviewers were adjudicated by a third investigator (A.A.) to reach consensus.

Data extraction was conducted independently by the same two reviewers using a standardised template [32]. Extracted variables included study design, sample size and population characteristics, geographical setting, publication year, article title, authorship and reported clinical outcomes.

### 2.3 Assessment of Risk of Bias

The methodological quality of the included studies was independently evaluated by two reviewers (D.M. and F.D.L.) using the Newcastle–Ottawa Scale (NOS; see Table S1) [33]. Each study was subsequently categorised as having a low, moderate or high risk of bias according to its total NOS score, following previously established approaches. Any discrepancies in scoring were resolved through consultation with a third reviewer (A.A.).

The following thresholds were applied: NOS scores < 4 denoted a high risk of bias, scores between 4 and 6 indicated a moderate risk and scores > 6 corresponded to a low risk of bias. The nine NOS domains assessed included representativeness of the exposed cohort, selection of the non‐exposed cohort, ascertainment of exposure, confirmation that the outcome was not present at baseline, comparability of cohorts (primary and additional factors), outcome assessment, adequacy of follow‐up duration and completeness of follow‐up.

According to these criteria, all included studies were deemed to present a low risk of bias, as detailed in Table S1.

## 3 Results

### 3.1 Findings From Systematic Search

Applying the predefined search strategy, a total of 1291 records were initially identified across PubMed (MEDLINE), Web of Science and Embase (via Ovid). After duplicate removal, 1241 unique articles were retained for screening. Titles and abstracts were evaluated against the eligibility criteria, resulting in the exclusion of 1202 studies that did not meet the inclusion requirements. The remaining 39 articles underwent full‐text assessment.

**FIGURE 1:**
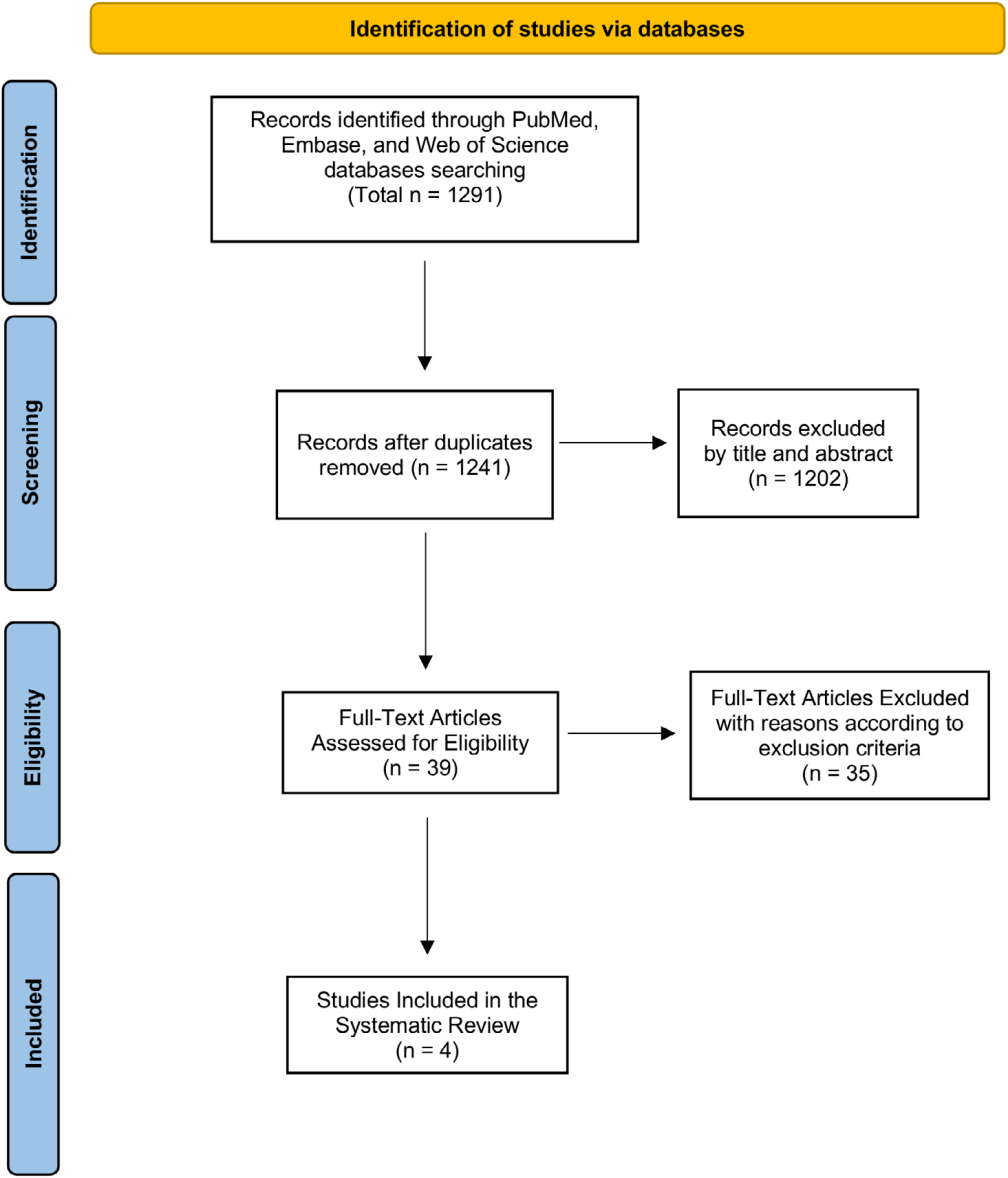
PRISMA flow diagram summarising the study selection process, from the initial identification of records through screening, eligibility assessment and final inclusion in the systematic review.

As illustrated in Figure 1, four studies satisfied all eligibility criteria and were ultimately included in the qualitative synthesis of this systematic review.

### 3.2 Description of Included Studies in the Systematic Review

In this systematic review four studies matched our review question and therefore considered the role of ABCB1/MDR1 polymorphism in relation to TMZ efficacy, and consequently, the survival in GB patients.

The first study, written by Schaich et al., is a clinical study involving 112 patients with GB treated with radiotherapy with or without concomitant/adjuvant TMZ [34]. Among the three ABCB1 SNPs evaluated, the C1236T polymorphism emerged as the only variant with a clear and clinically relevant association with outcome. Patients carrying the C/C genotype showed a markedly improved overall survival compared with both heterozygous (C/T) and homozygous mutant (T/T) individuals. In the subgroup treated with concomitant/adjuvant TMZ, the C/C genotype was associated with a two‐year overall survival of 37%, compared with only 8%–10% in the other genotype groups. This difference was further supported by multivariate analysis, in which the C/C genotype remained an independent predictor of better survival (HR ≈ 0.25 vs. C/T), indicating a strong genotype‐dependent modulation of TMZ efficacy. Mechanistically, the authors also observed genotype‐related differences in MDR1 expression, with the C/C carriers showing expression patterns consistent with lower P‐glycoprotein activity, potentially resulting in reduced efflux of TMZ and therefore increased intracellular drug exposure. Notably, the favourable effect of the C/C genotype was independent of MGMT promoter methylation, indicating that the benefit was not attributable to differences in the canonical DNA repair pathway but rather to ABCB1‐mediated pharmacokinetic mechanisms. Altogether, these observations provide a convincing indication that MDR1 genetic variability, specifically the exon 12 C1236T variant, can influence response to TMZ and support the potential value of ABCB1 genotyping in GB prognosis and treatment optimisation [34]. In addition to the C1236T variant, the authors also examined the other two major ABCB1 polymorphisms, G2677T and C3435T. Although both were associated with differences in MDR1 expression levels, neither showed a significant impact on survival outcomes, either in the overall cohort or among patients receiving TMZ‐based therapy. The survival curves for these genotypes were largely superimposable, suggesting that, within this study, the exon 12 polymorphism represents the only ABCB1 variant with clinically meaningful influence on TMZ responsiveness. Importantly, none of the three polymorphisms affected survival in patients treated with radiotherapy alone, reinforcing the conclusion that the observed effect of the C1236T SNP reflects a TMZ‐specific predictive role rather than an intrinsic prognostic factor. Overall, this study offers one of the first clinical indications that ABCB1‐mediated drug efflux may meaningfully contribute to inter‐individual variability in TMZ response [34].

Although the findings require validation in larger and more homogeneous cohorts, they highlight the potential value of integrating ABCB1 genotyping into future predictive models and raise the broader possibility that pharmacogenetic profiling of efflux transporters could help refine therapeutic stratification in GB [34].

The study by Oberstadt et al. focused on the epigenetic regulation and genetic variability of ABCB1 in GB, analysing whether promoter methylation or the common C3435T polymorphism influenced clinical outcome in a cohort of 64 patients [35]. Using a newly established pyrosequencing assay, the authors evaluated ABCB1 promoter methylation levels and found them to be significantly higher in GB tissue compared with non‐neoplastic brain. However, no association was observed between ABCB1 methylation and overall survival, either in the entire cohort or when considering only patients treated with TMZ. Furthermore, ABCB1 methylation did not correlate with ABCB1 mRNA expression, suggesting that methylation of this promoter region does not exert a functionally relevant regulatory effect [35]. The authors also genotyped the ABCB1 C3435T polymorphism, previously reported to affect P‐glycoprotein expression and activity. In this study, however, the C3435T variant showed no association with promoter methylation and no impact on patient survival, with survival curves essentially overlapping across genotype groups. These findings indicate that, within this cohort, neither epigenetic nor genetic variation of ABCB1 appeared to influence the clinical response of GB patients, including those treated with TMZ [35].

The results of Oberstadt et al. do not support a role for ABCB1 promoter methylation or the C3435T polymorphism as predictors of outcome in GB.

The study by Malmström et al. represents the largest and most methodologically rigorous effort to date examining the clinical relevance of ABCB1 single‐nucleotide variants (SNVs) in GB patients uniformly treated with radiotherapy concomitant with TMZ [36]. The authors evaluated four common ABCB1 variants, 1199G>A, 1236C>T, 2677G>T/A and 3435C>T, in an initial pilot cohort of 97 patients, with survival used as the primary outcome measure. In this first cohort, no association with overall survival was found for the synonymous variants 1236C>T, 2677G>T/A or 3435C>T, consistent with the negative findings reported in several other malignancies [36]. In contrast, the nonsynonymous 1199G>A variant initially appeared clinically relevant: patients carrying the heterozygous A/G genotype showed significantly poorer survival compared with G/G homozygotes (median OS 11.5 vs. 18.2 months, *p* = 0.012), and this association remained significant in multivariate analysis (HR ≈ 2.13). Given this signal, the authors expanded their analysis to a larger cohort of 179 primary GB (IDH‐wildtype) cases and performed an independent validation in an external confirmatory cohort. However, the initial survival disadvantage associated with the 1199A allele could not be replicated. In the expanded cohort, the difference between G/G and A/G + A/A no longer reached statistical significance (median OS 15.7 vs. 11.5 months, *p* = 0.085) and in the confirmatory Danish cohort the trend even reversed, with A/G carriers showing a non‐significant tendency toward better survival (13.8 vs. 16.8 months, *p* = 0.19). Multivariate analyses across cohorts consistently identified MGMT methylation, extent of surgery and age as major prognostic factors, while ABCB1 variants, including 1199G>A, were not independently associated with outcome [36]. Even when stratifying for MGMT methylation status, the authors found no evidence that ABCB1 SNVs modulate TMZ sensitivity in the subgroup of methylated tumours, those in which efflux‐mediated resistance would theoretically be most evident. While the initial signal for 1199G>A in the pilot dataset was intriguing, its disappearance in both the expanded and confirmatory cohorts underscores the importance of large, well‐controlled samples when evaluating pharmacogenetic markers [36]. So, these findings suggest that ABCB1 germline variation alone is unlikely to significantly shape TMZ response in clinical practice, and that other tumour‐intrinsic and microenvironmental factors may play a more dominant role in driving inter‐individual variability.

In the study by Munisamy et al., the impact of the ABCB1 1236C>T polymorphism was examined specifically in GB patients undergoing TMZ‐based chemoradiotherapy [37]. The GB subgroup consisted of 50 patients, all genotyped for the 1236C>T variant and evaluated for both TMZ pharmacokinetics and clinical outcome, while control group was composed by 150 subjects [37]. A clear genotype‐dependent pattern in plasma TMZ levels emerged: CC carriers exhibited the highest concentrations (5.11 ± 1.4 μg/mL), CT intermediate levels (3.59 ± 0.66 μg/mL) and TT the lowest (2.34 ± 1.3 μg/mL), with differences remaining statistically significant across metaboliser groups (*p* < 0.05). Despite this pharmacokinetic gradient, overall survival did not differ meaningfully between genotypes, with median OS values of 17.6 months for CC, 17.3 months for CT and 16.4 months for TT (*p* = 0.59). The authors noted that any survival trend related to the variant was confined mainly to CNS WHO grade 3 gliomas, while in GB the curves were essentially superimposable. When considering GB alone, these data indicate that although the ABCB1 1236C>T polymorphism significantly alters systemic TMZ exposure, it does not translate into a clinically relevant survival effect.

The absence of a genotype‐dependent outcome in GB aligns with the broader evidence suggesting that common ABCB1 variants, while pharmacologically plausible modifiers of drug efflux, do not reliably predict TMZ response or prognosis in GB patients [37]. A summary of the studies is provided in Table 3.

**TABLE 3:** Summary of studies included in this systematic review.

First author and year of publication	GB patients included (*n*)	Outcome	References
Schaich et al., 2009	*n* = 112	C/C genotype predicted improved 2‐year OS in TMZ‐treated patients, independent of MGMT methylation	[34]
Oberstadt et al., 2013	*n* = 64	Overall median survival: 459 days OS for patients treated with TMZ: 515 days OS for patients not treated with TMZ: 87 days The difference in survival between patients treated with and without TMZ was highly significant (*p* < 0.01).	[35]
Malmström et al., 2020	*n* = 179	Variants correlated with survival outcomes. The study suggests that the 1199G>A variant of the ABCB1 gene could be a new treatment predictor for GB patients treated with RT and TMZ	[36]
Munisamy et al., 2021	*n* = 50 *n* = 150 control patients	The polymorphism of the MDR1 gene (1236C>T) plays a role in plasma TMZ levels and also in the survival time of patients with glioma. Therefore, treatment with TMZ in malignant glioma can be predicted by genotyping the MDR1 SNP (1236C>T)	[37]

## 4 Discussion

Across solid tumours, clinical data indicate that functional variability in ABCB1 can influence chemotherapy response. Several prospective studies in lung and breast cancer cohorts show that common ABCB1 variants, particularly C1236T, C3435T and G2677T/A, associate with differences in survival and adverse‐event profiles under cytotoxic regimens [38]. In platinum‐treated lung cancer, heterozygous or variant genotypes in these loci have been associated with inferior overall survival compared with wild‐type carriers, suggesting genetically mediated modulation of effective drug exposure [38]. In breast cancer patients receiving anthracyclines and taxanes, C3435T genotypes have been linked to variability in non‐haematological toxicity, implying altered systemic handling and tissue distribution of ABCB1 substrates [39]. Experimental work further corroborates the causal plausibility: paclitaxel‐resistant Non‐small Cell Lung Cancer (NSCLC) cell lines acquire an ABCB1‐high phenotype, which is reversible by ABCB1 suppression, restoring intracellular drug accumulation and cytotoxicity [40].

Collectively, these converging clinical and mechanistic observations support the concept that ABCB1 activity is not a passive background variable, but a quantitative determinant of cytotoxic pharmacodynamics and that germline polymorphisms at core coding/linked loci can produce clinically meaningful variation in chemotherapeutic sensitivity.

Building on this evidence, we hypothesised that similar pharmacogenetic mechanisms could operate in GB (always referred as IDH‐wildtype CNS WHO grade 4), where therapeutic efficacy of TMZ critically depends on achieving sufficient intratumoral drug levels. In this context, ABCB1 represents a particularly relevant candidate determinant, given its strategic localisation at the luminal membrane of the blood–brain barrier and its capacity to actively extrude structurally diverse xenobiotics, including several alkylating agents. Although TMZ displays relatively high passive permeability compared to other chemotherapeutics, efflux transporters such as ABCB1 and ABCG2 are known to modulate central nervous system drug disposition, thereby potentially influencing both tumour exposure and systemic toxicity.

Despite this biologically plausible framework, clinical evidence linking ABCB1 polymorphisms to TMZ response in GB has remained fragmentary and inconsistent. Individual studies have been limited by small sample size, heterogeneity in genotyping platforms, variable endpoint definitions and lack of adjustment for major prognostic modifiers such as MGMT promoter methylation or IDH mutational status. Moreover, the contribution of ethnic background, a well‐documented determinant of ABCB1 allele frequency, has often been underexplored, further complicating the interpretation of cross‐study comparisons.

Accordingly, the rationale for the present systematic review was to synthesise and critically evaluate the available clinical data on ABCB1 genetic variability and its association with TMZ efficacy in GB. By integrating findings across independent cohorts, our goal was to clarify whether germline differences in ABCB1 might contribute to the interindividual variability observed in treatment outcomes and whether such polymorphisms could hold potential as predictive or prognostic biomarkers. Beyond their clinical implications, these analyses may also provide mechanistic insight into how BBB transport dynamics interface with chemotherapeutic pharmacogenomics in brain tumours, a domain still insufficiently characterised compared to non‐CNS malignancies.

When contrasted with studies reporting a significant effect of ABCB1 variants, such as the C1236T genotype in Schaich et al., these findings underscore the inconsistency across available datasets and highlight the need for larger, uniformly treated cohorts to clarify whether ABCB1‐mediated drug efflux meaningfully modulates TMZ sensitivity in clinical practice.

These results stand in contrast to the effect observed for C1236T in Schaich et al., and highlight that, at least in large TMZ‐treated GB cohorts, ABCB1 polymorphisms do not reliably predict survival. the work by Malmström et al. provides strong evidence that the most commonly investigated ABCB1 variants, including 1199G>A, 1236C>T, 2677G>T/A and 3435C>T, do not exert a clinically meaningful influence on outcome in GB patients receiving standard radiochemotherapy with TMZ.

Across the four studies included in this systematic review, encompassing a combined total of 405 GB patients and 150 healthy controls, the contribution of ABCB1 germline variability to TMZ response emerges as limited and markedly inconsistent. While the earliest evidence from Schaich et al. suggested a potentially meaningful association, identifying the ABCB1 C1236T C/C genotype as a robust determinant of improved survival in TMZ‐treated patients, independent of MGMT status, subsequent investigations failed to substantiate this signal. Neither the epigenetic analyses of Oberstadt et al., nor the large, methodologically rigorous cohorts examined by Malmström et al., nor the pharmacokinetic study by Munisamy et al. demonstrated a reproducible effect of ABCB1 variants on clinical outcome. Even in the presence of clear genotype‐dependent differences in systemic TMZ exposure, as observed for 1236C>T, survival curves in GB remained virtually superimposable, indicating that modulation of transporter activity does not meaningfully alter therapeutic benefit in this setting. These convergent negative findings are biologically plausible: although ABCB1 is strategically positioned at the luminal surface of the blood–brain barrier, TMZ's high passive permeability and the profound structural disruption of the barrier in GB diminish the relative contribution of efflux mechanisms to intratumoral drug delivery. Moreover, the modest functional impact of common ABCB1 polymorphisms is likely overshadowed by dominant molecular and clinical determinants of prognosis, including MGMT promoter methylation, IDH mutational status and extent of resection. Ethnic heterogeneity, underpowered cohort sizes and methodological variability across studies further attenuate the interpretability of isolated positive associations. Collectively, these data indicate that ABCB1 germline variation does not constitute a reliable biomarker of TMZ efficacy or survival in GB. Nevertheless, the consistent influence of the 1236C>T variant on plasma TMZ levels underscores the broader relevance of transporter pharmacogenomics and suggests that future work should move beyond single‐gene associations, integrating efflux transporter genetics with tumour‐intrinsic molecular features and spatial pharmacokinetics to refine our understanding of drug response in the central nervous system.

The evidence synthesised in this review benefits from several notable strengths. First, the included studies collectively span a range of methodological approaches, genotyping, epigenetic profiling, pharmacokinetic assessment and multivariate survival analysis, allowing a multidimensional evaluation of ABCB1 as a potential determinant of TMZ response in GB. The presence of two relatively large cohorts, particularly the 179‐patient dataset by Malmström et al., provides a degree of statistical robustness uncommon in the pharmacogenetic landscape of GB. Moreover, the only study incorporating TMZ plasma quantification (Munisamy et al.) offers mechanistic insight into how ABCB1 variability may shape systemic drug exposure, adding a valuable functional complement to purely association‐based analyses. Taken together, these features create a dataset that, while heterogeneous, captures both molecular and clinical dimensions of ABCB1‐mediated variability.

Nonetheless, the limitations of the current evidence are equally compelling. Across studies, sample sizes remain modest relative to the biological and clinical heterogeneity of GB and none are sufficiently powered to detect small effect sizes or genotype–environment interactions. Variability in study design, ranging from differences in genotyping platforms and analytical pipelines to inconsistent endpoint definitions and incomplete reporting of MGMT or IDH status, introduces potential confounding that cannot be fully resolved through narrative synthesis. Ethnic heterogeneity adds another layer of complexity, given the marked population‐specific differences in ABCB1 allele frequencies and linkage disequilibrium structures. The lack of harmonised pharmacokinetic data, aside from a single study, prevents a systematic exploration of how ABCB1 genotypes relate to intratumoral TMZ exposure, the parameter most relevant to therapeutic effect.

Furthermore, a relevant limitation of this review relates to the classification of GB in the included studies. Most cohorts were assembled before or around the implementation of the 2021 WHO classification of CNS tumours and therefore did not systematically incorporate IDH status into diagnostic definitions. As a consequence, a proportion of cases historically classified as GB may correspond to astrocytoma, IDH‐mutant, CNS WHO grade 4 according to current criteria. This potential misclassification introduces a source of heterogeneity and may have influenced survival analyses, given the distinct biological behaviour and more favourable prognosis associated with IDH‐mutant tumours.

Finally, the predominance of retrospective or observational designs limits causal inference and increases susceptibility to bias.

Together, these strengths and limitations highlight both the value and the fragility of the existing evidence base: while the available studies provide important mechanistic and clinical signals, they remain insufficient to support definitive conclusions regarding the predictive utility of ABCB1 polymorphisms in GB.

An additional consideration relates to the broader network of efflux transporters at the BBB. While the present analysis focused on ABCB1, other transporters, particularly ABCG2, are co‐expressed at the luminal interface and exhibit partially overlapping substrate specificity. Experimental and pharmacological evidence suggests that these transporters can act in a coordinated manner, with compensatory upregulation or functional redundancy limiting the impact of single‐transporter variability [27, 41]. Therefore, the lack of a consistent association between ABCB1 polymorphisms and clinical outcomes may, in part, reflect the contribution of parallel efflux systems not captured in the analysed studies. Future investigations integrating combined transporter genotypes and functional activity may provide a more comprehensive understanding of BBB‐mediated modulation of drug disposition in GB.

## 5 Conclusions and Future Perspectives

In this systematic review, we examined the clinical and mechanistic evidence linking ABCB1/MDR1 genetic variation to TMZ response in GB. Across four independent studies involving more than 400 GB patients, the collective findings indicate that common ABCB1 polymorphisms exert, at most, a modest and inconsistent influence on therapeutic outcome. Only one study reported a significant survival advantage associated with the C1236T C/C genotype, whereas the remaining investigations, spanning promoter methylation analyses, multivariate survival modelling and pharmacokinetic profiling, did not corroborate a clinically meaningful role for ABCB1 in modulating TMZ efficacy. Although genotype‐dependent differences in systemic TMZ exposure were observed, particularly for 1236C>T, these pharmacokinetic effects did not translate into improved survival in GB, suggesting that efflux‐mediated modulation at the BBB is overshadowed by dominant tumour‐intrinsic and microenvironmental factors.

Looking forward, these findings underscore the need for a more integrated approach to pharmacogenomic biomarker discovery in GB.

Future studies should incorporate harmonised genomic, epigenomic and pharmacokinetic datasets, evaluated within sufficiently powered, treatment‐uniform cohorts and stratified by key molecular features such as MGMT methylation, IDH mutational status and tumour evolutionary dynamics. Mechanistically informed efforts, combining transporter genomics with spatial pharmacology, BBB imaging and intratumoral drug quantification, will be essential to determine whether efflux transporters contribute to therapeutic resistance in ways not captured by germline variation alone. Ultimately, advancing the precision medicine framework in GB will require moving beyond single‐gene analyses toward multidimensional models capable of capturing the complex interplay between drug delivery, tumour biology and host pharmacogenomics.

Furthermore, although previous generations of systemic ABCB1 inhibitors failed largely because of toxicity and insufficient specificity, the therapeutic potential of modulating efflux activity at the tumour–BBB interface should not be dismissed. Emerging technologies, such as nanoparticle‐based drug carriers, aptamer‐guided delivery and RNA‐based targeting, now offer the possibility of selectively attenuating ABCB1 function within the tumour microenvironment while sparing its physiological roles in systemic detoxification. These approaches provide a more refined path forward, suggesting that ABCB1 modulation, if applied with spatial precision, could still enhance drug accumulation and potentiate the efficacy of alkylating agents like TMZ. Therefore, future work integrating these targeted strategies with molecular stratification of patients may ultimately define whether ABCB1 can be converted from a theoretical barrier into a therapeutically actionable vulnerability in GB.

## Author Contributions

Conceptualisation, E.E. and A.A.; methodology, D.M. and M.D.; data curation, D.M. and F.D.L.; writing – original draft preparation, A.A. and F.D.L.; writing – review and editing, A.P.C., G.Ci. and G.Ca.; supervision, E.E. and G.Ca. All authors have read and agreed to the published version of the manuscript.

## Funding

The authors have nothing to report.

## Ethics Statement

The authors have nothing to report.

## Consent

The authors have nothing to report.

## Conflicts of Interest

The authors declare no conflicts of interest.

## Supporting information


**File S1:** Table with risk of bias judgements according NOS score.

## Data Availability

All data generated or analysed during this study are included in this published article and its Supporting Information files.
