# Supplementary material for: ABCB1 Polymorphisms Influence on Temozolomide Resistance and Overall Survival in Glioblastoma Patients: A Systematic Review of Clinical Evidence
Source: J Cell Mol Med. 2026 Apr 9;30(7):e71130. doi: 10.1111/jcmm.71130 (PMC13065491; doi:10.1111/jcmm.71130)
Supplement: Supplementary file 1 — File S1: Table with risk of bias judgements according NOS score. [file JCMM-30-e71130-s001.pdf]

RISK OF BIAS Table	SELECTION				COMPARABILITY		OUTCOME			
	Representative of the exposed cohort	Selection of external control	Ascertainment of exposure	Outcome of interest not present at the start of the study	Comparability of Cohorts		Assessment of outcomes	Sufficient follow-up time	Adequacy of follow-up	
					Main Factor	Additional Factor				
	**1**	**2**	**3**	**4**	**5**	**6**	**7**	**8**	**9**	**OVERALL**
**Schaich et al., 2009**	X		X		X	X	X	X	X	7
**Oberstadt et al., 2013**	X		X		X	X	X	X	X	7
**Malmström et al., 2020**	X		X		X	X	X	X	X	7
**Munisamy et al., 2021**	X	X	X		X	X	X	X	X	8

Supplementary File 1. Table with Risk of Bias judgements according NOS score
